# Putative sex-specific human pheromones do not affect gender perception, attractiveness ratings or unfaithfulness judgements of opposite sex faces

**DOI:** 10.1098/rsos.160831

**Published:** 2017-03-08

**Authors:** Robin M. Hare, Sophie Schlatter, Gillian Rhodes, Leigh W. Simmons

**Affiliations:** 1 Centre for Evolutionary Biology, School of Animal Biology, The University of Western Australia, Crawley, Western Australia 6009, Australia; 2 ARC Centre of Excellence in Cognition and its Disorders, School of Psychology, The University of Western Australia, Crawley, Western Australia 6009, Australia

**Keywords:** pheromones, mate choice, attractiveness, humans, androstadienone, estratetraenol

## Abstract

Debate continues over the existence of human sex pheromones. Two substances, androstadienone (AND) and estratetraenol (EST), were recently reported to signal male and female gender, respectively, potentially qualifying them as human sex pheromones. If AND and EST truly signal gender, then they should affect reproductively relevant behaviours such as mate perception. To test this hypothesis, heterosexual, Caucasian human participants completed two computer-based tasks twice, on two consecutive days, exposed to a control scent on one day and a putative pheromone (AND or EST) on the other. In the first task, 46 participants (24 male, 22 female) indicated the gender (male or female) of five gender-neutral facial morphs. Exposure to AND or EST had no effect on gender perception. In the second task, 94 participants (43 male, 51 female) rated photographs of opposite-sex faces for attractiveness and probable sexual unfaithfulness. Exposure to the putative pheromones had no effect on either attractiveness or unfaithfulness ratings. These results are consistent with those of other experimental studies and reviews that suggest AND and EST are unlikely to be human pheromones. The double-blind nature of the current study lends increased support to this conclusion. If human sex pheromones affect our judgements of gender, attractiveness or unfaithfulness from faces, they are unlikely to be AND or EST.

## 1. Introduction

Chemical communication is arguably the oldest and most common form of communication among living things [1]. Chemical signals used by animals for intraspecific communication are called pheromones [2]. Invertebrates such as arthropods use pheromones to broadcast aggregation, alarm, territorial or sex information to conspecifics [3–5]. Sex pheromones in particular invoke varied but predictable responses by informing recipients about the broadcaster's species, sex, mating status (i.e. mated or unmated) and mate quality [1,6]. In comparison, vertebrate animals are behaviourally complex, and their responses to pheromones are not as straightforward. Because of this complexity, there is some debate about whether vertebrates use pheromones *sensu stricto*. Some experts argue new terms should be used that account for the complex responses to chemical signals vertebrates evince [7], whereas others argue that the original definition should be expanded [8]. For the purposes of this paper, we use the following definition of pheromone: ‘any substance that is released by an individual…that promotes subsequent variation in the…behaviour of individuals within the same species’ [1, p. 267].

Among mammals, sex pheromones may variously indicate oestrus (which would otherwise remain cryptic), build parent–offspring bonds, modify pubertal processes, terminate pregnancy in the presence of unfamiliar males or delineate territorial boundaries [9–11]. In some cases, sex pheromones are used as cues of sperm competition. Male meadow voles (*Microtus pennsylvanicus*) exposed to male-specific pheromones during mating increase sperm allocation even in the absence of competing males [12]. In humans (*Homo sapiens*), assessments of mate sex or quality involve relatively complex cognitive processes. However, considering the phylogenetic placement of humans among taxa that use pheromones to communicate sexual information, it seems reasonable to expect that humans might also use pheromones to some degree when identifying and assessing potential mates. Both sexes report that body scent affects sexual interest [13], and women value olfactory cues more than visual cues when choosing partners [14], but no unambiguous human sex pheromones have been identified to date.

The best known candidates for human sex pheromones are the steroids androstadienone (AND; associated with men) and estratetraenol (EST; associated with women), identified and reported by a corporation in 1991 [15]. Men produce AND in the axillae and testes, and it is a component of sweat and semen [16]. The same steroid appears in the urine of women, reportedly in smaller quantities than in men, and is synthesized in the third trimester of pregnancy into EST [16,17]. The gender-specific nature of the steroids remains controversial and is yet to be rigorously demonstrated [18]. Indeed, recent research indicates AND has identical effects on the attractiveness of vocal signals in both genders [19]. Given this controversy, as well as problems with definitions of pheromones, concerns about how to design experiments testing the effects of AND and EST, and the confidential nature of the protocols and data of the commercial assay that uncovered the steroids, critics argue there are no grounds to assume that AND and EST are human pheromones [15,20–22]. Despite the lack of evidence establishing AND and EST as pheromones [15], a body of human pheromone research has grown around the steroids, reporting various effects on behaviour, physiology and mood [23–25].

Of these studies, two provide modest evidence for pheromonal functions of AND and EST. Saxton *et al*. [26] showed that women exposed to AND in a speed-dating setting sometimes reported higher attraction to potential mates than women exposed to a control scent. Zhou *et al*. [27] exposed participants to AND, EST or a control scent while showing them short animations of gender-neutral walk cycles. Heterosexual male participants were more likely to assign femaleness to the stimuli when exposed to EST, whereas heterosexual female and homosexual male participants were more likely to assign maleness to the stimuli when exposed to AND [27]. By demonstrating sex-specific effects on mate assessment and perceptions of gender, these results comprise the strongest evidence to date that the steroids might function as human sex pheromones.

This research aimed to extend these findings by examining the effects of the putative sex pheromones on perceptions of facial gender, attractiveness and unfaithfulness. Faces are particularly important in mate choice, signalling attractiveness by honestly indicating genetic diversity, physical health and immunocompetence [28–30]. Humans also use faces to judge sexual faithfulness [31], albeit with limited accuracy. Women are slightly more accurate than chance at assessing an unknown male face for unfaithfulness [32], and men are slightly better than chance at selecting the more unfaithful of two women [33]. If AND and EST are indeed human pheromones, then they should not only signal gender but also influence reproductively relevant behaviours, such as perceptions of a potential mate's attractiveness or unfaithfulness.

The predictions of our research were threefold. First, we predicted that participants presented with a gender-neutral face, created by morphing male and female faces together, should attribute maleness to the stimulus if exposed to AND or femaleness if exposed to EST. Such a finding would extend Zhou *et al*.'s [27] findings from body perception to face perception, thus increasing the evidence that AND and EST signal gender. Second, we predicted that participants would rate the faces of potential mates differently for attractiveness when exposed to AND or EST as opposed to a control scent. Specifically, faces should appear more attractive when presented with the corresponding-gender chemical because of reinforcement from olfactory and visual modalities, or if the chemicals signal mate value, by indicating health or sexualization. In these scenarios, faces paired with the opposite-gender pheromone should appear less attractive because of contradictory or ambiguous gender information received via olfactory and visual modalities.

Our third prediction was that differences caused by exposure to AND or EST might extend to ratings of predicted unfaithfulness in potential mates. If these substances provide cues to potential reproductive competition, faces might appear more unfaithful when presented with the opposite-gender pheromone, as the opposite-gender pheromone might be interpreted as evidence of recent or frequent mating.

We tested these expectations using two computer-based tasks. The first task was intended to extend Zhou *et al*.'s [27] findings that AND and EST cause gendered perception of gender-neutral stimuli. The second task was intended to expand on these findings by examining the effects of AND and EST on the perceived attractiveness and unfaithfulness of potential mates.

## 2. Materials and methods

### 2.1. Participants and task design

Self-reported heterosexual, non-smoking, adult Caucasian participants (43 male and 51 female; mean age = 23.7; s.d. age = 5.8) were recruited from the University of Western Australia campus. The first task tested perceptions of gender, and the second task assessed perceptions of attractiveness and unfaithfulness. Of the 94 participants, 46 completed the gender task, and 94 completed the two rating tasks. To control for individual differences in ratings, each participant completed the same tasks twice, on consecutive days, and received different olfactory stimuli on each day. Participants were assigned one of the two pheromone treatments (AND or EST, masked with clove oil) pseudo-randomly, to ensure balance, and every participant received the control (clove oil only). Stimulus order (treatment first or second) was randomized to counterbalance any effects of learning or familiarity. Treatments were administered by a cotton ball taped under the nose, so that participants were exposed to the assigned stimulus throughout each of the computer sessions. Testing took place in June–September 2015 under a male experimenter (R.M.H.; 48 participants) and March–July 2016 under a female experimenter (S.S.; 46 participants). Both tasks were displayed and recorded on a Macintosh computer (screen size = 21.5 inches; resolution = 1920 × 1080 pixels; operating system = OS X version 10.8.5).

**Figure 1.**
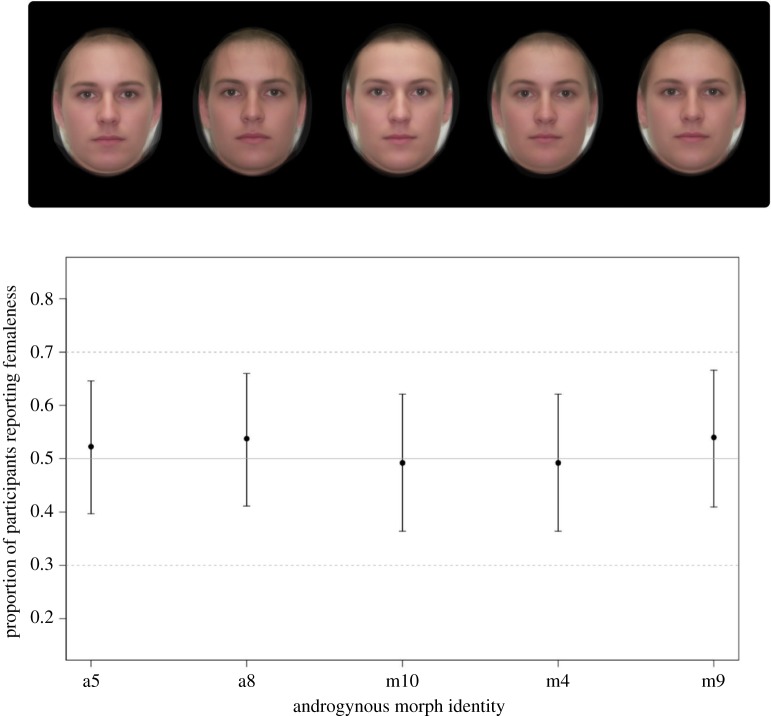
Five gender-neutral face morphs used in the gender classification task. Eighty-two participants were no more likely to assign femaleness to face morphs than would be expected by chance (proportion assigning female with 95% CIs from binomial tests).

The first task, lasting approximately 2–5 min, excluding a 2 min buffer for the treatment to take effect, showed participants five gender-neutral morphed faces (figure 1) for 2 s each and required the participant to assign a gender, either male or female, to each face. The proportion of faces perceived as female was recorded on each day, and a difference score was created for each participant by subtracting scores in the control condition from scores in the treatment condition. Each gender-neutral morph in this task was created by morphing five male and five female faces taken from the image set used in the ratings task (below), in Abrosoft FantaMorph Deluxe version 5.4.5. When choosing faces from the dataset for morphing, bald or bearded men were excluded to prevent distinctly gendered visual cues appearing in the morphs. Other than this stipulation, faces were selected randomly from the dataset. Prior to their use in the task, gender neutrality of the face morphs was confirmed in a pilot study by asking 82 (32 male) participants (mean age = 30.3; s.d. age = 12.1) whether the face morphs were male or female. Participants were no more likely to assign femaleness to a face than by chance alone (figure 1).

The second task, lasted approximately 10–25 min and continued directly from the first task. Participants were shown front-view colour photographs of opposite-sex Caucasian adult faces with neutral expressions for 2 s each. Oval masks hid most of the hair of the faces, leaving visible the face contour and inner hairline. Face images measured 320 × 420 pixels and were displayed at 72 pixels per inch. Female participants viewed 102 male faces (mean age of photographic models = 24.7; s.d. age = 6.9), and male participants viewed 86 female faces (mean age of photographic models = 24.5; s.d. age = 5.8). The photographs were taken from the same database used by Rhodes *et al*. [32]. Participants recorded attractiveness and predicted unfaithfulness scores, each on a scale from 1 (low) to 10 (high), for each face. Difference scores (treatment minus control) for average attractiveness and average unfaithfulness were created for each participant. Contrary to previous studies [34], experimenter sex had no effect on our results (for attractiveness, *F*_1,92_ = 0.94 and *p* = 0.336; for unfaithfulness, *F*_1,92_ = 0.09 and *p* = 0.765).

### 2.2. Steroid acquisition and preparation

AND and EST were purchased from Steraloids, Inc. (USA). Following the procedure in Zhou *et al*. [27], 500 µM AND was suspended in 1% v/v clove oil propylene glycol solution (4 ml) for the ‘male’ treatment, and 500 µM EST was suspended in 1% v/v clove oil propylene glycol solution (4 ml) for the ‘female’ treatment (clove oil is commonly used to mask and control for any detectable scents, and to ensure treatments are indistinguishable by scent alone). The control treatment consisted of 1% v/v clove oil propylene glycol solution (4 ml). To ensure objectivity, a confederate mixed and assigned coded labels to the treatments, and the researchers remained blind to the identity of the treatment stimuli throughout testing and statistical analyses of data. Treatment containers were stored at −80°C. On testing days, the required treatment containers were taken from the freezer approximately 45 min before the first test to thaw before testing.

### 2.3. Statistical analysis

Analyses were performed in R version 3.1.2 [35]. All statistical models used in the analyses were tested for normality of residuals using Shapiro–Wilk tests for distribution [36] and Breusch–Pagan tests for equal variance [37]. RESET tests were performed to ensure appropriate model form [38]. Shapiro–Wilk tests were performed using the function built into R, and Breusch–Pagan and RESET tests were performed using the *lmtest* package [39]. In the case of models failing normality tests, the *sandwich* package was used to create robust covariate matrices for analysis [40]. Effect sizes (*r*) and 95% confidence intervals (CIs) for the factors of interest were also calculated [41]. The identity of the putative pheromones used in each treatment was revealed subsequent to statistical analysis.

## 3. Results

### 3.1. Gender classification task

**Figure 2.**
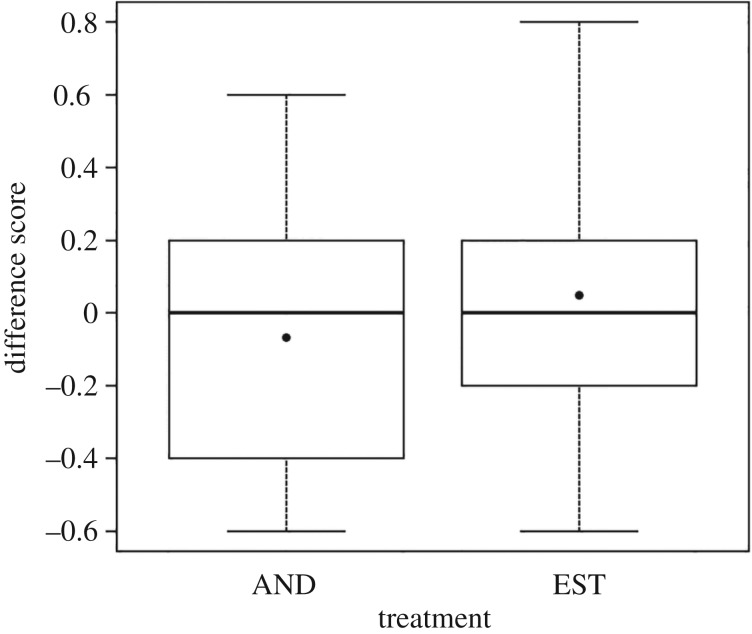
Mean difference scores generated in the gender classification task from 46 participants exposed to one of two putative human pheromones, AND and EST. Differences calculated for each participant by subtracting control from treatment proportions (proportion of five gender-neutral faces perceived as female on separate days). Thick lines indicate medians, boxes indicate interquartile ranges, whiskers indicate minima and maxima, and points indicate means. A positive difference score would indicate that participants were more likely to attribute femaleness in the treatment setting than in the control setting.

The proportion of faces perceived as female in control and treatment sessions was calculated for each participant. A difference score was calculated for each participant by subtracting the control from the treatment proportion. Thus a difference score other than zero would indicate an effect of the putative pheromones on gender perception. A positive difference score would indicate the participant was more likely to attribute femaleness when exposed to the pheromone treatment, and a negative difference score would indicate the participant was more likely to attribute maleness when exposed to the pheromone treatment. A consistent positive difference score among participants exposed to EST would thus support our prediction that the substance signals femaleness. A consistent negative difference score among participants exposed to AND would support our prediction that AND signals maleness. Difference scores from all participants were pooled and analysed with three factors: pheromone (AND or EST), participant sex (FEMALE or MALE) and stimulus order (treatment FIRST or SECOND), including an interaction between pheromone and sex. Order was not a factor of interest but was included in the model to counterbalance any potential effects of learning in the experimental protocol or acquired familiarity with the stimulus set. ANOVA indicated no significant effects of any factor on difference scores (figure 2 and table 1).

**Table 1.** ANOVA of difference scores of the proportion of faces perceived as female for five gender-neutral morphs, including effect sizes (*r*) and 95% confidence intervals (CIs). (Differences calculated by subtracting control from treatment data. Asterisk denotes interaction between factors.)

factor	d.f.	*F*-value	*p*-value	*r*	lower CI	upper CI
pheromone	1	0.827	0.369	0.141	−0.156	0.414
sex	1	0.029	0.866	0.027	−0.266	0.315
order	1	2.464	0.124	0.238	−0.056	0.494
pheromone*sex	1	0.393	0.534	0.097	−0.199	0.377
residuals	41					

### 3.2. Attractiveness ratings

**Figure 3.**
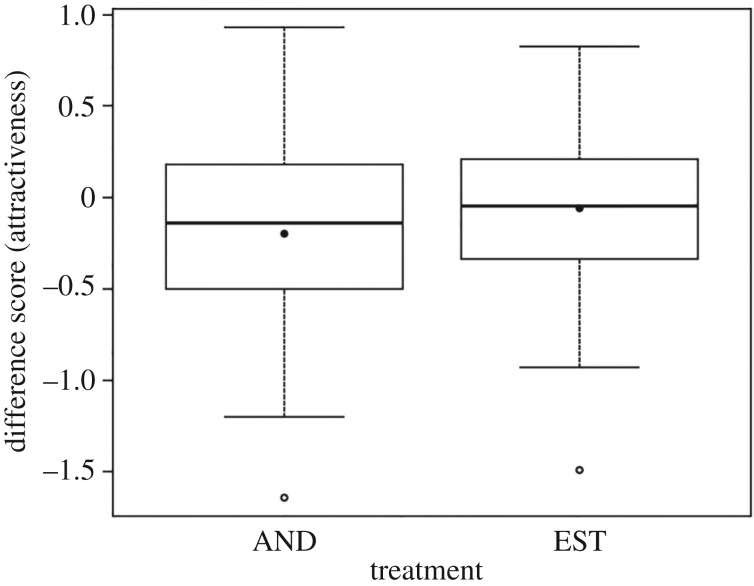
Difference scores generated in the attractiveness rating task from 94 participants exposed to one of two putative human pheromones, AND and EST. Differences calculated by subtracting control from treatment ratings. Thick lines indicate medians, boxes indicate interquartile ranges, whiskers indicate minima and maxima, dark points indicate means and open points indicate outliers. A positive difference score would indicate participants found faces more attractive in the treatment setting than in the control setting.

Difference scores were calculated for each participant by subtracting the average attractiveness rating when exposed to the control stimulus from the average rating when exposed to the treatment stimulus. Deviations from a mean difference of zero would thus indicate an effect of the treatment on attractiveness ratings. A positive difference score among participants exposed to matching-gender face and pheromone stimuli would support our prediction that AND and EST enhance attractiveness. As with the gender classification data, three between-participants factors were used: pheromone, order and participant sex, with an interaction between pheromone and sex. ANOVA indicated no significant effects (figure 3 and table 2) of participant sex or pheromones on the difference scores. There was a significant procedural effect of order; while there was no difference in ratings between treatment and control scents when the treatment (either AND or EST) was administered on the first day (difference score mean = 0.088, s.d. = 0.39), participants exposed to the treatment on the second day recorded negative difference scores (difference score mean = −0.31, s.d. = 0.50; table 2). This procedural effect had no bearing on our predictions and was excluded from further consideration.

**Table 2.** ANOVA of difference scores in the attractiveness ratings, including effect sizes (*r*) and 95% confidence intervals (CIs). (Differences calculated by subtracting control from treatment (AND or EST) mean for each participant. Asterisk denotes interaction between factors.)

factor	d.f.	*F*-value	*p*-value	*r*	lower CI	upper CI
pheromone	1	2.351	0.129	0.160	−0.044	0.352
sex	1	1.695	0.196	0.137	−0.068	0.330
order	1	17.083	<0.001	0.401	0.216	0.559
pheromone*sex	1	1.056	0.307	0.108	−0.096	0.304
residuals	89					

### 3.3. Unfaithfulness ratings

**Figure 4.**
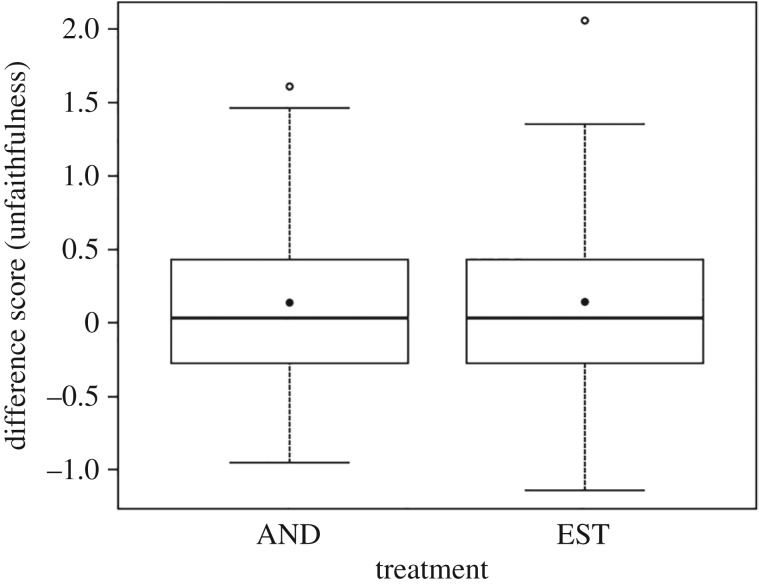
Difference scores generated in the unfaithfulness rating task from 94 participants exposed to one of two putative human pheromones, AND and EST. Differences calculated by subtracting control from treatment ratings. Thick lines indicate medians, boxes indicate interquartile ranges, whiskers indicate minima and maxima, solid points indicate means and open points indicate outliers. A positive difference score would indicate participants found faces more likely to be unfaithful in the treatment setting than in the control setting.

Difference scores were taken for each participant by subtracting the average control unfaithfulness rating from the average treatment unfaithfulness rating. Again, a deviation from zero would indicate an effect of the putative pheromones. Our prediction that AND and EST affect judgements of unfaithfulness would be supported in the case that participants exposed to mismatching-gender of face and pheromone stimuli recorded positive difference scores (i.e. more unlikely to be unfaithful in a relationship). Three between-participants factors were used: pheromone, order and participant sex, with an interaction between pheromone and sex. ANOVA indicated no significant effects of pheromone, sex or order on the difference scores recorded (figure 4 and table 3).

**Table 3.** ANOVA of difference scores in the unfaithfulness ratings, including effect sizes (*r*) and 95% confidence intervals (CIs). (Differences calculated by subtracting control from treatment (AND or EST) mean for each participant. Asterisk denotes interaction between factors.)

factor	d.f.	*F*-value	*p*-value	*r*	lower CI	upper CI
pheromone	1	0.002	0.965	0.005	−0.198	0.207
sex	1	0.137	0.712	0.039	−0.165	0.240
order	1	0.202	0.654	0.048	−0.157	0.248
pheromone*sex	1	0.322	0.572	0.060	−0.144	0.260
residuals	89					

## 4. Discussion

The hypotheses that exposure to the putative pheromones AND and EST would: (i) alter gender perceptions of gender-neutral stimuli, and (ii) alter perception of the attractiveness and probable unfaithfulness of potential mates were not supported. While consistent with studies like that of Ferdenzi *et al*. [19], these results contrast with those of other studies showing that AND affects perceptions of attractiveness [26] and that AND and EST affect perceptions of gender [27]. There are several potential reasons why AND and EST did not have an effect in our study, falling into two categories: either our experimental design prohibited accurate determination of the substances' effects or the substances do not act as signals of gender or of mate value, in which case they do not qualify as sex pheromones.

It is unlikely that problems with our experimental design interfered with measurements of the effects of AND and EST. Our treatment concentrations were identical to those deployed by Zhou *et al*. [27], far exceeding concentrations present in human armpits but matching the standard set by pioneering studies of AND and EST [42] and used in virtually all studies of the chemicals. Other aspects of our experiment (e.g. repeated measures, exposure to gender-neutral stimuli) broadly matched those of Zhou *et al*. [27]. Other aspects of the experimental design are equally unlikely to have driven the discrepancy. It has been reported that AND and EST take effect within minutes and are potent for at least one hour [43], and participants were alone for the duration of the testing period, so any chemicals secreted by the researchers were unlikely to have interfered with the experiment. Differences in experimental protocol between this study and that of Zhou *et al*. [27] are unlikely to explain the discrepancy in results between our studies. Nonetheless, we recognize that our gender assignment task was relatively underpowered, given the small number of stimuli used [44].

The alternative explanation, that AND and EST are not sex pheromones, is more plausible. Although its association with pregnancy automatically qualifies EST as a sex-specific substance, it is possible that EST may not actively signal femaleness, instead signalling other information such as prior mating or is pregnant; it may alternatively be a simple metabolic by-product with no signalling function. Similarly, because AND is produced by both men and women, albeit in different quantities, the substance might have no sex-specific signalling functions, in which case one might expect that exposure to AND would have no substantial effects on judgements of gender or of potential mates. That higher concentrations of AND are not perceived as more pleasant than lower concentrations [23] adds weight to this conclusion. Finally, the prevalence of specific anosmia to androstenes in both men and women (at rates up to 37%) casts doubt on the evolutionary use of substances such as AND and EST in mate choice scenarios [23]. The pregnancy-specific nature of EST, the gender-ambiguous nature of AND and the prevalence of androstene anosmia might explain why exposure to AND and EST did not alter perceptions of gender, attractiveness or unfaithfulness, but do not explain the discrepancy in results between this study and those cited above.

One important strength of our study was blind data recording. Experimenters aware of treatment identities may be predisposed unconsciously to record data confirming their expectations or else unconsciously to predispose participants to do the same [45]. A recent movement has called for improved scientific reporting to reduce the incidence of this unconscious bias [45–48]. Accordingly, the researchers of this study remained blind to the identity of the treatments (AND and EST) until after statistical analysis was complete. This removed the potential for unconscious bias in the recording of results, and prevented the researchers from predisposing participants to record biased ratings. The blind nature of data collection and analysis in our study lends increased weight to our conclusion that AND and EST do not function as human pheromones.

Following Wyatt [15], we recommend a return to first principles in the search for human pheromones, and recommend scientific rather than commercial bioassays of human chemical output as an unbiased method of identifying true human pheromones. It is possible that AND and EST have functions, pheromonal or otherwise, unrelated to mate assessment, and it is likely there are other chemicals secreted by humans that are currently unknown but that may function as pheromones. Critical replication of reported effects of AND and EST, as well as research into the functions of other putative human pheromones, is recommended; however, if any human chemicals affect our judgements of gender, attractiveness or unfaithfullness from faces, they are unlikely to include AND or EST.

## Data accessibility

Data and R script available from Research Data Online @ UWA [49].

## References

[1] Johansson BG, Jones TM 2007 The role of chemical communication in mate choice. Biol. Rev. 82, 265–289. (doi:10.1111/j.1469-185X.2007.00009.x) 17437561 10.1111/j.1469-185X.2007.00009.x

[2] Karlson P, Lüscher M 1959 ‘Pheromones’: a new term for a class of biologically active substances. Nature 183, 55–56. (doi:10.1038/183055a0) 13622694 10.1038/183055a0

[3] Birch M, Haynes K 1982 Insect pheromones. London, UK: Edward Arnold.

[4] Wyatt TD 2009 Pheromones and other chemical communication in animals. In Encyclopedia of neuroscience (ed. Squire LR), pp. 611–616. Oxford, UK: Academic Press.

[5] Wyatt TD 2015 How animals communicate via pheromones. Am. Sci. 103, 114–121. (doi:10.1511/2015.113.114)

[6] Thomas ML 2011 Detection of female mating status using chemical signals and cues. Biol. Rev. 86, 1–13. (doi:10.1111/j.1469-185X.2010.00130.x) 20233168 10.1111/j.1469-185X.2010.00130.x

[7] Wyatt TD 2009 Fifty years of pheromones. Nature 457, 262–263. (doi:10.1038/457262a) 19148082 10.1038/457262a

[8] Wysocki CJ, Preti G 2009 Pheromones in mammals. In Encyclopedia of neuroscience (ed. Squire LR), pp. 625–632. Oxford, UK: Academic Press.

[9] Bronson F et al. 1983 Pheromones and reproduction in mammals. San Diego, CA: Academic Press.

[10] Gosling LM, Roberts SC 2001 Scent-marking by male mammals: cheat-proof signals to competitors and mates. Adv. Stud. Behav. 30, 169–217. (doi:10.1016/S0065-3454(01)80007-3)

[11] Hurst JL, Beynon RJ, Roberts SC, Wyatt TD 2008 Chemical signals in vertebrates 11. New York, NY: Springer.

[12] delBarco-Trillo J, Ferkin MH 2004 Male mammals respond to a risk of sperm competition conveyed by odours of conspecific males. Nature 431, 446–449. (doi:10.1038/nature02845) 15386011 10.1038/nature02845

[13] Thornhill R, Gangestad SW 1999 The scent of symmetry: a human sex pheromone that signals fitness? Evol. Hum. Behav. 20, 175–201. (doi:10.1016/S1090-5138(99)00005-7)

[14] Havlicek J, Saxton TK, Roberts SC, Jozifkova E, Lhota S, Valentova J, Flegr J 2008 He sees, she smells? Male and female reports of sensory reliance in mate choice and non-mate choice contexts. Pers. Indiv. Differ. 45, 565–570. (doi:10.1016/j.paid.2008.06.019)

[15] Wyatt TD 2015 The search for human pheromones: the lost decades and the necessity of returning to first principles. Proc. R. Soc. B 282 (doi:10.1098/rspb.2014.2994) 10.1098/rspb.2014.2994 PMC4375873 25740891

[16] Gower DB, Ruparelia BA 1993 Olfaction in humans with special reference to odorous 16-androstenes: their occurrence, perception and possible social, psychological and sexual impact. J. Endocrinol. 137, 167–187. (doi:10.1677/joe.0.1370167) 8326246 10.1677/joe.0.1370167

[17] McClintock MK 2009 Pheromones in humans and social chemosignals. In Encyclopedia of neuroscience (ed. Squire LR), pp. 617–623. Oxford, UK: Academic Press.

[18] Wyatt TD 2014 Pheromones and animal behavior: chemical signals and signatures. Cambridge, UK: Cambridge University Press.

[19] Ferdenzi C, Delplanque S, Atanassova R, Sander D 2016 Androstadienone's influence on the perception of facial and vocal attractiveness is not sex specific. Psychoneuroendocrinology 66, 166–175. (doi:10.1016/j.psyneuen.2016.01.016) 26827295 10.1016/j.psyneuen.2016.01.016

[20] Winman A 2004 Do perfume additives termed human pheromones warrant being termed pheromones? Physiol. Behav. 82, 697–701. (doi:10.1016/j.physbeh.2004.06.006) 15327919 10.1016/j.physbeh.2004.06.006

[21] Pause BM 2004 Are androgen steroids acting as pheromones in humans?. Physiol. Behav. 83, 21–29. (doi:10.1016/j.physbeh.2004.07.019) 15501487 10.1016/j.physbeh.2004.07.019

[22] Doty RL 2014 Human pheromones: do they exist? In Neurobiology of chemical communication (ed. Mucignat-Caretta C), pp. 535–360. Boca Raton, FL: CRC Press/Taylor, Francis.

[23] Havlicek J, Murray AK, Saxton TK, Roberts SC 2010 Chapter three: current issues in the study of androstenes in human chemosignaling. In Vitamins, hormones (ed. Gerald L), pp. 47–81. Burlington, VT: Academic Press. 10.1016/S0083-6729(10)83003-1 20831942

[24] Mostafa T, Khouly GE, Hassan A 2012 Pheromones in sex and reproduction: do they have a role in humans? J. Adv. Res. 3, 1–9. (doi:10.1016/j.jare.2011.03.003)

[25] Semwal A, Kumar R, Teotia UVS, Singh R 2013 Pheromones and their role as aphrodisiacs: a review. J. Acute Dis. 2, 253–261. (doi:10.1016/S2221-6189(13)60140-7)

[26] Saxton TK, Lyndon A, Little AC, Roberts SC 2008 Evidence that androstadienone, a putative human chemosignal, modulates women's attributions of men's attractiveness. Horm. Behav. 54, 597–601. (doi:10.1016/j.yhbeh.2008.06.001) 18601928 10.1016/j.yhbeh.2008.06.001

[27] Zhou W, Yang X, Chen K, Cai P, He S, Jiang Y 2014 Chemosensory communication of gender through two human steroids in a sexually dimorphic manner. Curr. Biol. 24, 1091–1095. (doi:10.1016/j.cub.2014.03.035) 24794295 10.1016/j.cub.2014.03.035

[28] Rhodes G 2006 The evolutionary psychology of facial beauty. Annu. Rev. Psychol. 57, 199–226. (doi:10.1146/annurev.psych.57.102904.190208) 16318594 10.1146/annurev.psych.57.102904.190208

[29] Little AC, Jones BC, DeBruine LM 2011 Facial attractiveness: evolutionary based research. Phil. Trans. R. Soc. B 366, 1638–1659. (doi:10.1098/rstb.2010.0404) 21536551 10.1098/rstb.2010.0404 PMC3130383

[30] Perrett DI, Lee KJ, Penton-Voak I, Rowland D, Yoshikawa S, Burt DM, Henzi SP, Castles DL, Akamatsu S 1998 Effects of sexual dimorphism on facial attractiveness. Nature 394, 884–887. (doi:10.1038/29772) 9732869 10.1038/29772

[31] Berry DS 1990 Taking people at face value: evidence for the kernel of truth hypothesis. Soc. Cogn. 8, 343–361. (doi:10.1521/soco.1990.8.4.343)

[32] Rhodes G, Morley G, Simmons LW 2013 Women can judge sexual unfaithfulness from unfamiliar men's faces. Biol. Lett. 9, 1–6. (doi:10.1098/rsbl.2012.0908) 10.1098/rsbl.2012.0908 PMC3565506 23221873

[33] Leivers S, Simmons LW, Rhodes G, McCormick CM 2015 Men's sexual faithfulness judgments may contain a kernel of truth. PLoS ONE 10, e0134007 (doi:10.1371/journal.pone.0134007) 26244776 10.1371/journal.pone.0134007 PMC4526544

[34] Jacob S, Hayreh DJS, McClintock MK 2001 Context-dependent effects of steroid chemosignals on human physiology and mood. Physiol. Behav. 74, 15–27. (doi10.1016/S0031-9384(01)00537-6) 11564447 10.1016/s0031-9384(01)00537-6

[35] R Core Team. 2014 R: a language and environment for statistical computing. Vienna, Austria: R Foundation for Statistical Computing.

[36] Royston JP 1982 The W test for normality. J. R. Stat. Soc. C, Appl. 31, 176–180. (doi:10.2307/2347986)

[37] Breusch TS, Pagan AR 1979 A simple test for heteroscedasticity and random coefficient variation. Econometrica 47, 1287–1294. (doi:10.2307/1911963)

[38] Ramsey JB 1969 Tests for specification errors in classical linear least-squares regression analysis. J. R. Stat. Soc. B Met. 31, 350–371. (doi:10.2307/2984219)

[39] Zeileis A, Hothorn T 2002 Diagnostic checking in regression relationships. R News 2, 7–10.

[40] Zeileis A 2004 Econometric computing with HC and HAC covariance matrix. J. Stat. Softw. 11, 1–17. (doi:10.18637/jss.v011.i10)

[41] Nakagawa S, Cuthill IC 2007 Effect size, confidence interval and statistical significance: a practical guide for biologists. Biol. Rev. 82, 591–605. (doi:10.1111/j.1469-185X.2007.00027.x) 17944619 10.1111/j.1469-185X.2007.00027.x

[42] Lundström JN, Hummel T, Olsson MJ 2003 Individual differences in sensitivity to the odor of 4,16-androstadien-3-one. Chem. Senses 28, 643–650. (doi:10.1093/chemse/bjg057) 14578126 10.1093/chemse/bjg057

[43] Jacob S, McClintock MK 2000 Psychological state and mood effects of steroidal chemosignals in women and men. Horm. Behav. 37, 57–78. (doi:10.1006/hbeh.1999.1559) 10712859 10.1006/hbeh.1999.1559

[44] Westfall J, Kenny DA, Judd CM 2014 Statistical power and optimal design in experiments in which samples of participants respond to samples of stimuli. J. Exp. Psych. Gen. 143, 2020–2045. (doi:10.1037/xge0000014) 10.1037/xge0000014 25111580

[45] Ioannidis JPA, Munafò MR, Fusar-Poli P, Nosek BA, David SP 2014 Publication and other reporting biases in cognitive sciences: detection, prevalence, and prevention. Trends Cogn. Sci. 18, 235–241. (doi:10.1016/j.tics.2014.02.010) 24656991 10.1016/j.tics.2014.02.010 PMC4078993

[46] Simmons JP, Nelson LD, Simonsohn U 2011 False-positive psychology: undisclosed flexibility in data collection and analysis allows presenting anything as significant. Psychol. Sci. 22, 1359–1366. (doi:10.1177/0956797611417632) 22006061 10.1177/0956797611417632

[47] Kühberger A, Fritz A, Scherndl T, Fanelli D 2014 Publication bias in psychology: a diagnosis based on the correlation between effect size and sample size. PLoS ONE 9, e105825 (doi:10.1371/journal.pone.0105825) 25192357 10.1371/journal.pone.0105825 PMC4156299

[48] Holman L, Head ML, Lanfear R, Jennions MD 2015 Evidence of experimental bias in the life sciences: why we need blind data recording. PLoS Biol. 13, e1002190 (doi:10.1371/journal.pbio.1002190) 26154287 10.1371/journal.pbio.1002190 PMC4496034

[49] Hare RM, Schlatter S, Rhodes G, Simmons LW 2017 Data from: Putative sex-specific human pheromones do not affect gender perception, attractiveness ratings or unfaithfulness judgements of opposite sex faces. Research Data Online @ UWA. See https://researchdataonline.research.uwa.edu.au/handle/123456789/3401. 10.1098/rsos.160831 PMC5383829 28405372

